# The Reproductive Transition: Effects on Viral Replication, Immune Activation, and Metabolism in Women with HIV infection

**DOI:** 10.1007/s11904-021-00594-7

**Published:** 2021-12-08

**Authors:** Rebecca Abelman, Phyllis C. Tien

**Affiliations:** 1 Department of Medicine, University of California, San Francisco, CA 94143 USA; 2 Medical Service, Department of Veteran Affairs Medical Center, San Francisco, CA 94121 USA; 3 San Francisco VAMC, Infectious Disease Section, University of California, 111W 4150 Clement Street, CA 94121 San Francisco, USA

**Keywords:** Women, Menopause, Aging, HIV replication, Immune activation, Metabolic parameters

## Abstract

**Purpose of Review:**

To describe research advances in the menopausal transition (MT) and its effects on HIV replication, immune activation, and metabolic parameters in women living with HIV (WLWH).

**Recent Findings.:**

Physiologic changes due to declines in ovarian reserve characterize the MT. Evidence suggests that estrogen depletion influences HIV replication and the latent reservoir. Changes in markers of immune activation, waist circumference, and neurocognition, independent of chronologic age, occur before the final menstrual period (FMP). HIV effects on gut microbial translocation and adipose tissue, as well as health disparities in WLWH may contribute. Improved biomarker sensitivity to predict FMP provides opportunities to study MT in WLWH.

**Summary:**

Research is needed to determine the effects of MT and HIV on virologic and clinical outcomes, using accurate assessments to predict the FMP and menopausal stages. These findings could inform the timing of interventions to prevent early onset of adverse outcomes in WLWH.

## Introduction

The extended survival afforded by effective antiretroviral therapy (ART) has enabled women with HIV to live well into their postmenopausal years. Over half of people diagnosed with HIV in the United States (US) [1] and an estimated nearly one quarter of people with HIV globally are now over 50 years of age [2]. As the population of women living with HIV (WLWH) ages, there has been growing attention on the menopausal transition and the accompanying changes in sex steroid levels on HIV replication, immune activation and inflammation, and alterations in metabolic parameters.

Menopause results from the complete depletion of ovarian follicles and is associated with the loss in ovarian production of sex steroids including estrogen, progesterone, and androgens, and the cessation of menses [3]. The menopausal transition is a multi-year transition period generally referred to as perimenopause or the time period between pre- and postmenopause [4]. The perimenopause period is characterized physiologically by compensatory changes in ovarian and pituitary hormone production due to declines in ovarian reserve (i.e., the number of follicles remaining in the ovary), irregular menstrual cycles, and in some women, overt vasomotor symptoms [3]. During this period, alterations in immune function and inflammatory responses, and metabolic parameters have also been reported to occur, with menopause serving as a well-known risk factor for clinical outcomes such as cardiovascular disease, osteoporosis, and neurocognitive changes. These immune and metabolic perturbations, and its clinical sequelae are especially relevant to WLWH, as HIV infection itself has been associated with gut microbial translocation, immune activation and systemic inflammation, and metabolic perturbations, which have been implicated in the early onset of clinical outcomes such as cardiovascular disease and low bone density [5–10]. Recent studies suggest that estrogen depletion with ovarian aging modulates the immune response to HIV, including the activity and size of the HIV reservoir. Taken together, for WLWH, changes in metabolic parameters, immune function, and inflammation associated with both HIV infection and menopause could lead to adverse health outcomes, and is of significant clinical concern. Understanding the additional effects of estrogen depletion in WLWH could inform the optimal timing of interventions to slow down the longer term clinical sequelae of HIV infection.

This review will address the advances in assessing the menopausal transition, its effects on immune activation and inflammation, and metabolic parameters, and implications in HIV research and clinical care.

### The Menopausal Transition and Challenges in its Assessment

Disentangling the effects of chronologic aging and ovarian aging has stymied our understanding of the effects of the menopausal transition on clinical outcomes. Quantifying ovarian aging as precisely as possible has been a major goal of investigators working in this area. In 2001 [11], the Stages of Reproductive Aging Workshop (STRAW) formalized a consensus staging criteria for the reproductive life cycle in women that has evolved over the past two decades with advances in research and development of sensitive biomarkers to predict the final menstrual period (FMP). The menopausal transition follows the reproductive stage and is comprised of an early and late transition phase that is defined by self-reported menstrual cycle patterns [4]. Entry into the early phase occurs when there is a 7-day or more difference in length of consecutive cycles, while the late phase is defined by a woman having an interval of amenorrhea ≥ 60 days (that is not related to chronic illness or treatments that might affect the menstrual cycle). Vasomotor symptoms are more likely to be present in the late phase, which is thought to commence 1 to 3 years before the FMP, marking the entry into early menopause [4].

The reported menstrual irregularities are a result of compensatory changes in the hypothalamic-pituitary-ovarian axis. The loss of ovarian follicular reserve with aging leads to declines in estradiol and inhibin B, a nonsteroidal hormone produced by larger, growing follicles during the follicular phase of the menstrual cycle. The loss of the negative feedback by inhibin B consequently leads to an increase in follicle-stimulating hormone (FSH) secretion from the pituitary. Compensatory changes occur to preserve estradiol levels, which at times are associated with levels of estradiol that are even higher than in the reproductive phase. Eventually, compensatory mechanisms fail and FSH becomes more consistently elevated, while estradiol becomes more consistently low. The reduced follicular quality is also accompanied by declines in progesterone production during the luteal phase of the menstrual cycle leading to anovulatory cycles, and ultimately the cessation of menses.

HIV infection adds an additional layer of difficulty in determining menopausal status and ovarian follicular reserve. Amenorrhea (or irregular menses) and abnormal symptoms are common due to chronic illness and have made determination of the menopausal transition and FMP difficult. A systematic review of the age at menopause in WLWH found a range from 46 to 50 years depending on the study [12]. In the general population of US women, menopause is reported to occur at a median age of 51 years [13]. While STRAW + 10 [4] included the addition of biomarkers such as FSH, inhibin B, and anti-Mullerian hormone (AMH) to self-reported changes in menstrual cycle pattern, as supporting criteria to stage the menopausal transition, FSH and inhibin B may be harder to interpret in WLWH who are more likely to have irregular menstrual cycles. Furthermore, greater body mass index (BMI), which is especially prevalent in WLWH [14], can affect FSH levels. A multi-site study of racially and ethnically diverse women enrolled from the general population found that not all women experienced one pattern of FSH rise or estradiol decline over the menopausal transition, and that obese women of all racial groups tended to have a lower FSH level at the time of stabilization followed by higher postmenopausal estradiol levels as compared to non-obese women [15]. Another study from the Ms. cohort of mostly African-American and Hispanic women with and without HIV who had a history of substance use found that opiate use was associated with lower FSH, and greater BMI with lower inhibin B levels [16]. In that study, biomarkers were measured during the early follicular phase of the menstrual cycle, i.e., within the first days of menses [16]. While rigorous sampling of biomarkers during the early follicular phase of the menstrual cycle has been employed in epidemiological studies, it is not optimal in women such as those with HIV who are more likely to have irregular menstrual cycles due to chronic illness. Reliance on markers such as FSH could lead to misclassification of women, if FSH is used alone to determine menopausal status.

AMH, a biomarker of growing interest, is produced by granulosa cells of small, growing follicles in the ovary and is a direct indicator of functional ovarian reserve [17]. Serum AMH levels have been strongly correlated with the number of growing follicles and can be tested from specimens obtained without regard to menstrual phase timing, although there may be some intra-cycle variation [17]. AMH usually becomes undetectable during late perimenopause, and is thought to be a useful predictor of time to FMP [3]. Improved sensitivity and automation of AMH assays has strengthened its use as a preferred biomarker of ovarian reserve [18]. However, studies show that factors such as use of hormonal contraceptives and BMI may influence levels [17]. A study from the Women’s Interagency HIV Study (WIHS) also found that CD4 count in both women with and without HIV infection affected AMH levels, suggesting that CD4 cells may influence the function of ovarian follicles [19]. Further study of the use of AMH and the STRAW + 10 self-reported criteria to accurately assess the time to FMP is needed.

### Estrogen, immune function, and HIV viral replication

Estrogen, and specifically β-estradiol, the active form of estrogen, affects immune function including modulation of T cell subsets and the innate immune responses [20]. In vitro models suggest that β-estradiol can enhance the ability of plasma dendritic cells (pDCs) to respond to toll-like receptor 7 (TLR7) stimulation as a result of viral infection [21]. pDCs recognize HIV single-stranded RNA through TLR7 and secrete interferon and other inflammatory cytokines to promote antiviral activity [22]. The major effects of estradiol are mediated through estrogen receptor (ER) α and ERβ, both of which are expressed in immune and mucosal epithelial cells [23]. ER has been shown to be a negative regulator of the latent reservoir [24••]. Both estradiol and progesterone are thought to inhibit HIV replication in CD4 + T cells and macrophages [25, 26]. Indeed, a study of women with HIV not on antiretroviral therapy showed that HIV RNA levels fluctuate during the menstrual cycle with lower HIV RNA levels during the luteal phase of the menstrual cycle when estradiol levels rise [27].

Until recently, few studies examining biological sex differences on HIV reservoirs have taken into account menopausal status in women. A cross-sectional study of biological sex differences on the HIV latent reservoir in virally suppressed adults performed a sub-analysis that examined 11 postmenopausal and 11 pre-menopausal women and did not find a measurable difference in the frequency of inducible replication-competent HIV using a quantitative viral outgrowth assay [22]. In that study, women had a median age of 45 years (interquartile range: 40, 54), so some pre-menopausal women may have been in the perimenopausal transition when fluctuating and declining estradiol levels are occurring. Another recent study that examined resting T cells isolated from 14 virally suppressed WLWH at multiple time points across the menopausal transition (pre-, peri, and postmenopause) found significantly higher levels of inducible HIV RNA + cells using a sequencing-based assay in postmenopausal women compared to peri- and premenopausal women, and in perimenopausal women compared to premenopausal women [28••]. These results suggest that estrogen depletion with aging affects the immune response to HIV including the activity and size of the HIV reservoir. Further research is needed using larger sample sizes of women with accurate determination of the menopausal transition to better understand biological sex differences.

### HIV and Menopausal Transition and Effects on Microbial Translocation and Immune Activation

Both HIV infection and estrogen depletion have been associated with increased gut permeability and subsequent immune activation and inflammation. A pathway by which HIV infection is reported to be associated with immune activation is through persistent disruption of gut epithelial integrity and microbial translocation as a result of depletion of gut mucosal CD4 + lymphocytes early in the course of HIV infection [5, 8]. Sex steroid deficiency has been associated with gut permeability and immune activation in murine models [29]. Estradiol has an important role in repair and regeneration of mucosal surfaces, including the gut, genitourinary, and respiratory mucosa. Estrogen depletion may therefore impair gut mucosal function and increase microbial translocation and inflammation [30]. Estrogen depletion has been shown to increase secretion of the proinflammatory cytokines interleukin (IL)-1, IL-6, and tumor necrosis factor (TNF)-α in peripheral blood mononuclear cell monocytes [31]. In vitro studies also show that estrogen repletion lowers cytokine levels [23]. Much of our knowledge about sex steroid effects on immune function have been through in vitro studies.

Two recent longitudinal studies in women provide evidence that the menopausal transition is associated with increases in gut permeability and immune activation. A small study in 65 US women without HIV infection from the Study of Women’s Health Across the Nation (SWAN) found that fatty acid–binding protein 2, a marker of gut epithelial integrity, and soluble CD14 (sCD14), a marker of immune activation resulting from gut microbial translocation, increased in stored blood samples collected from 3 to 5 years after the FMP when compared to samples collected from 3 to 5 years prior to the FMP [32]. In an exploratory analysis, they also showed that the increase in gut permeability over the menopausal transition was associated with higher levels of CRP and lower bone mineral density. The other study in 350 US women with HIV infection from the WIHS found higher levels of plasma sCD14 and another marker of immune activation sCD163 in postmenopausal women compared to pre-menopausal women [33••]. A notable finding was that the sCD14 trajectory was significantly steeper in the period from 1 year before the FMP to 1–2 years after the FMP, and appeared to plateau after 2 years. Their results suggest that immune activation resulting from gut microbial translocation rises during the time period spanning the late perimenopause phase to early menopause when complete depletion of ovarian reserve occurs. Interestingly, menopausal status was not associated with markers of gut epithelial integrity and systemic inflammation, including intestinal fatty acid–binding protein, IL-6, and TNF-receptor 1, contrary to prior small studies in women without HIV that examined similar markers of inflammation [34, 35]. Whether estrogen depletion augments the heightened state of microbial translocation and systemic inflammation in the setting of HIV infection needs study, especially as higher levels of markers of intestinal fatty acid binding protein and systemic inflammation have been associated with mortality in people living with HIV [36]. Accurate assessment of markers of estrogen depletion are also needed in epidemiologic studies of WLWH.

Changes in gut microbiome composition have also been postulated to occur as a result of the menopausal transition. A study of 432 women with and without HIV also in the WIHS found a significant difference in overall gut microbiome composition between pre-menopausal (defined as reporting a normal or slightly irregular menstrual cycle in the past 12 months) and postmenopausal women who reported not having a menstrual period in the past 12 months, but only in WLWH [37]. The mechanism behind this difference is not clear. They originally hypothesized that a difference in overall gut microbiome composition by menopausal status could be explained by not only the reported effects of estrogen on gut mucosal repair [38], but also the metabolism of estrogen in the gut by bacteria via glucuronide deconjugation [39]. However, they did not find that menopausal status was associated with the bacterial beta-glucuronidase pathway. Their finding of a lack of statistical difference by menopausal status in the 151 women without HIV infection is also contrary to prior gut microbiome studies in women [40–42], but those studies were of even smaller sample size and were conducted in non-US settings. Geography, dietary patterns, as well as obesity have been shown to impact the gut microbiota [43]. Variability in sex steroid levels between and within groups could also explain differences.

Future studies are needed to longitudinally examine gut microbiome composition in WLWH and women without HIV infection across the pre-, peri-, and postmenopausal phases, and the relative contributions of mucosal disruption due to HIV and/or estrogen depletion. Assessing the relationship of HIV infection, estrogen depletion, gut microbial translocation, and markers of immune activation to clinical outcomes is imperative. Examination of a broader array of associated markers of systemic inflammation may also be warranted in the context of HIV infection and estrogen depletion.

### Menopausal Transition and Metabolic Perturbations

**Fig. 1:**
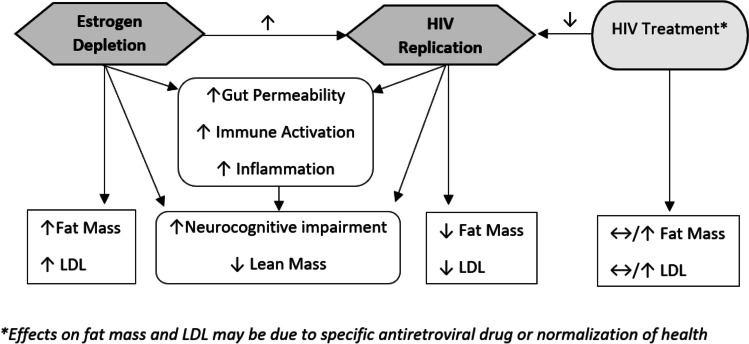
Proposed pathways by which estrogen depletion and HIV affects virologic, immune, and metabolic parameters

Metabolic perturbations that arise during the menopausal transition have been reported in women without HIV infection. HIV infection itself and its treatment have also been associated with metabolic perturbations including adiposity changes and dyslipidemia. An understanding of the menopausal transition on metabolic parameters in WLWH is critical. Figure 1 provides proposed pathways by which estrogen depletion and HIV might influence metabolic parameters. A longitudinal study of 1246 women in SWAN followed from 8 years before through 10.5 years after the FMP found an acceleration in increasing fat mass and declining proportion of lean mass during the menopausal transition defined as the 2 years prior to and after the FMP [44]. The acceleration then stabilized beyond this period. A notable finding was that weight and BMI climbed steadily but a menopausal transition-related acceleration was not observed. A longitudinal study in the WIHS using AMH to stage the menopausal transition, suggested that HIV infection blunts the trajectory of increase in BMI over the menopausal transition, whereas the expected trajectory of increase in waist circumference, a marker of visceral obesity, is largely preserved [45]. The blunting of the BMI increase in WLWH could be partly explained by HIV-associated depletion in subcutaneous adipose tissue mitochondrial DNA and mitochondrial dysfunction [46]. The SWAN study also demonstrated an accelerated increase in LDL-C in the 1-year period before and after the FMP with levels then plateauing [47]. Despite the eventual plateau in metabolic parameters, they found that greater increases in LDL-C within 1 year of the FMP were associated with greater odds of carotid plaque, a marker of atherosclerotic disease [48]. The effects of HIV infection, effective antiretroviral therapy, and specific antiretroviral agents which have been associated with metabolic perturbations including adiposity and lipoprotein changes need study during the menopausal transition.

The contribution of the menopausal transition to longer term clinical sequelae in WLWH is also emerging. A large study in WLWH in the WIHS that categorized women as being premenopausal, early perimenopausal, late perimenopausal, and in menopause using the STRAW criteria [11] demonstrated cognitive declines over 6 years in the learning, memory, and attention/working memory domains from the premenopause to the early perimenopause, and from the premenopause to postmenopause [49••]. That study also found that cognitive declines in learning and memory persisted in menopause, unlike prior studies in SWAN [50, 51], suggesting that other factors faced by WLWH in the WIHS, including low education, substance use, and viral hepatitis coinfection may play a role in the persistence of neurocognitive impairment after the onset in the perimenopause. Another small study of 30 women with and without HIV infection found that postmenopausal women had greater intramyocardial triglyceride content measured by cardiac MRI than premenopausal women, and intramyocardial triglyceride content in perimenopausal women was intermediate, suggesting a possible gradation in risk of diastolic dysfunction [52]. That study categorized menopausal stage using both self-report of menstrual cycle pattern and AMH levels. These studies provide insight into the perimenopausal period as a potential window for interventions to prevent longer term clinical outcomes.

## Conclusion

The menopausal transition in women marks a period of physiologic changes due to declines in ovarian production of estrogen and is increasingly recognized in WLWH to influence the HIV latent reservoir and viral replication. There is also a growing body of published studies in women with and without HIV infection that suggest a possible accelerated increase in markers of gut microbial translocation, immune activation and inflammation, and metabolic parameters during the menopausal transition. Greater research is needed to determine the effects of HIV as well as psychosocial stressors and health disparities that confront WLWH on these alterations during the menopausal transition. The use of biomarkers to accurately predict the FMP is critically needed to inform the timing of interventions to prevent the early onset of adverse HIV and clinical outcomes. Additionally, standardized criteria to define the menopausal stages need to be established in epidemiologic studies to accurately determine the effects of accelerated increases during the menopausal transition on longer term clinical outcomes.
